# Repercussions of the COVID-19 pandemic on child and adolescent mental health: A matter of concern—A joint statement from EAP and ECPCP

**DOI:** 10.3389/fped.2022.1006596

**Published:** 2022-11-28

**Authors:** L. Reali, R. G. Nijman, A. Hadjipanayis, S. Del Torso, P. Calamita, I. Rafele, M. Katz, S. Barak, Z. Grossman

**Affiliations:** ^1^ Primary Care Pediatrician, Italian National Health System (INHS), ASL Rm1, Rome, Italy; ^2^ Department of Pediatric Emergency Medicine, Division of Medicine, St. Mary's Hospital - Imperial College NHS Healthcare Trust, London, United Kingdom; ^3^ Section of Pediatric Infectious Diseases, Department of Infectious Diseases, Faculty of Medicine, Imperial College London, London, United Kingdom; ^4^ Centre for Pediatrics and Child Health, Imperial College London, London, United Kingdom; ^5^ European Society of Emergency Paediatrics, European Society of Emergency Medicine, Brussels, Belgium; ^6^ European Academy of Paediatrics (EAP), Brussels, Belgium; ^7^ Medical School, European University Cyprus, Nicosia, Cyprus; ^8^ Department of Paediatrics, Larnaca General Hospital, Larnaca, Cyprus; ^9^ ChildCare WorldWide—CCWWItalia OdV, Padova, Italy; ^10^ Primary Care Pediatrician, Italian National Health System (INHS), ASL Rm 6, Rome, Italy; ^11^ Primary Care Pediatrician, Italian National Health System (INHS), ASL Rm 3, Rome, Italy; ^12^ Patient Safety Department, Meuhedet Health Services, Tel Aviv, Israel; ^13^ Dana Dwek Children's Hospital, Tamsc, Tel Aviv, Israel; ^14^ Department of Pediatrics, Adelson School of Medicine, Ariel University Pediatrics, Ariel, Israel; ^15^ Department of Pediatrics, Maccabi Health Care Services Pediatrics, Tel Aviv, Israel

**Keywords:** anxiety, depression, lockdown, pandemic, stress, SARS-CoV-2, psychological impact, syndemic

## Abstract

COVID-19 pandemic and the consequent rigid social distancing measures implemented, including school closures, have heavily impacted children's and adolescents' psychosocial wellbeing, and their mental health problems significantly increased. However, child and adolescent mental health were already a serious problem before the Pandemic all over the world. COVID-19 is not just a pandemic, it is a syndemic and mentally or socially disadvantaged children and adolescents are the most affected. Non-Communicable Diseases (NCDs) and previous mental health issues are an additional worsening condition. Even though many countries have responded with decisive efforts to scale-up mental health services, a more integrated and community-based approach to mental health is required. EAP and ECPCP makes recommendations to all the stakeholders to take action to promote, protect and care for the mental health of a generation.

## The pandemic

The World Health Organization (WHO) declaration on March 11, 2020 that the disease caused by the severe acute respiratory syndrome coronavirus 2 (SARS-CoV-2) was an ongoing global pandemic and it forced almost all countries in Europe, and all over the world, to implement rigid social distancing measures, including strict lockdowns and school closures, to limit the transmission of the infection in the community. These measures heavily impacted children and adolescents' psychosocial wellbeing and, as a result, the incidence of sleep disturbances, anxiety, mood disorders, depression, eating problems, mental health problems and suicide (ideation, attempts or deaths) has significantly increased almost globally (1–9).

Leading experts on child health, including the American Academy of Pediatrics, declared a national state of emergency in child and adolescent mental health in October 2021 (10).

This crisis falls upon an already serious problem, that began emerging way before the Pandemic broke, of rising child and adolescent mental issues. In fact, in 2019 the estimated prevalence of mental disorders for boys and girls aged 10–19, was 16.3 per cent, while the global figure for the same age group was 13.2 per cent in the same year. This means that more than 9 million adolescents aged 10–19 in Europe lived with a mental disorder (11).

## The cost of mental disorders in Europe

Before the COVID-19 outbreak, poor mental health related issues costed the EU 4% of gross domestic product (GDP) in lost productivity and social expenses (12). We need to utilize effective strategies to strengthen families to respond, care, and protect a future for their children (11) and to promote a comprehensive and strong mental health system policy response (11) for adults and an increasing prevalence of mental conditions in young people (10–24 years) has been observed across several European countries between 1990 and 2019 (13), at the present, this situation has worsened (11). If the problem is not urgently addressed, through cost-effective interventions (14), this pandemic may have long term adverse consequences on children's and adolescents' mental health (15), as well as on the world economy, because these children grow into adults affected by mental health issues (16). In 2022 mental disorders account for at least 18% of global disease burden, and the associated annual global costs are projected to be US$ 6 trillion by 2030 (17). Hence, identifying cost-effective interventions is important for effective mental health care allocation, while is also essential that the intervention strategies are effective in reducing the burden of disease within the constraints of the allocated resources (18).

## A syndemic

Moreover, COVID-19 is not just a pandemic, it is a syndemic, defined as: “Two categories of disease interacting within specific populations—infection with severe acute respiratory syndrome coronavirus 2 (SARS-CoV-2) and an array of non-communicable diseases (NCDs). The syndemic nature of the threat we face means that a more nuanced approach is needed if we are to protect the health of our communities” (19). Most of the studies reported that the stress that children and adolescents have been subjected to, due to school closures and social distancing during the pandemic, impacted their mental health producing negative and different side effects (20–23), depending on their age, gender, ethnicity, family circumstances, socioeconomic situation (22, 24). Greater negative effects on any pre-existing mental health problem (25) and heavier mental health outcomes were found in those who already had poor mental health before the COVID-19 pandemic (26, 27). Furthermore, countries' health outcomes were influenced by local political decisions and media intervention, following public reactions, allowing for a major difference in morbidity and mortality within and between countries.

The mental health impacts on children and adolescent of differing policies may have different impacts. German children and adolescents presented more mental health and quality of life problems after the COVID-19 pandemic (24), even though the incidence and mortality rates in Germany were low when compared to countries such as Spain, Italy or China, or Portugal and their lockdown measures were less severe than in these countries (24, 28).

Therefore, our approach should be to analyze and intervene on this pandemic as “individual countries syndemics”, to emphasize local influences that determined better or worse outcomes, when comparing with other countries (29).

## Children and adolescent response to the pandemic is linked to their parents' support and the mentally or socially disadvantaged ones are the most affected

Beyond morbidity, pandemics carry secondary impacts, such as children orphaned or bereft of their caregivers. Such children often face adverse consequences, including poverty, abuse, and institutionalization (30).

Children's psychological response to the outbreak is strongly related to how their parents experienced the events (31) during the pandemic, especially if exacerbated by financial and material hardship (32). On the other hand, caregivers' coping and caregiver–child relationships are strongly related to children's and adolescents' emotional and behavioral functioning (33, 34).

Overall, stress levels in caregivers decreased over time, but stress specifically related to caregiving responsibilities worryingly continued to increase (35). Altogether, a close caregiver–child relationship was protective (36) and conducive for strengthening family relationships (37, 38); whereas, conversely, a strained caregiver–child relationship led to high-conflict interactions owing to increased exposure to stress, maltreatment, and depression within families (39–41). Therefore, if depression or anxiety in a child or adolescent are suspected, family and caregivers' roles and wellbeing are crucial (6, 7, 42, 43).

Mental health problems worsened especially in children and adolescents who already had a disorganized attachment or were affected by pre-existing behavioral problems like autism and Attention Hyperactivity Disorder (ADHD), not only immediately after stress exposure but even later. Adolescents, particularly, were more likely to experience high rates of depression, stress, and anxiety during and after pandemic, with increased substance abuse, suicide, relationship problems and school problems (44, 45). Moreover, robust evidence shows that high levels of stress (toxic stress) can influence the psychophysical development of children and adolescents, and the highest levels of stress (toxic stress) typically occur in low-income families (15).

Preexisting suffering from Non-Communicable Diseases (NCDs), as diabetes, chronic respiratory diseases, cardiovascular diseases, obesity, or neurodevelopmental issues proved to be an additional worsening condition (46, 47). NCDs are a massive contributor to COVID-19 mortality and severe illness across all age groups. The response to coronavirus has constrained children and adolescents with NCDs and severely disrupted their access to essential services, furthermore they are more likely to live in more financially stressed families and may have been more vulnerable to additional financial stress as a result of the pandemic (47).

## How the educational services answered to the children and adolescents needs during pandemic

Despite the attempt to maintain educational services through online tools, mental health support for young people was substantially discontinued. However, schools are not just places where students develop and progress their academic skills (48, 49): the daily routine and social interactions that they experience there can help them maintain good mental health. Moreover, the role of the school, in some countries as United States (50), or France (51) is even wider, covering not only primary needs but also representing a preventive reference point as well to identify conditions of maltreatment and neglect. Closing the schools determined a twofold negative effect, firstly, it prevented the identification of prolonged abuse and neglect, secondly, it lenghthen the exposure to these conditions. Thus, school closures have substantially contributed to the weakening of protective factors (52–54), particularly for young people from disadvantaged backgrounds (26, 55) and not all young people have been comfortable using digitally enabled services for support (56). Moreover, schools and universities can offer additional opportunities to modify NCD risk factors, favoring primary prevention across the life-course and improving knowledge of NCDs impact, promoting healthy behaviors and policies related to correct nutrition and physical activity, with the aim of thwarting obesity and diabetes, and control tobacco and alcohol use (57). Therefore, school closure and rigid social distancing measures for a long time should be avoided (1–3, 6, 45–47, 53).

## The way forwards

National and regional health policies should be based on evidence-based, effective strategies, tailored to children and young people's specific needs shared with local researchers and experts in the field (7, 11, 53, 57, 58). Even though many countries have responded with decisive efforts to scale-up mental health services, a more integrated and community-based approach to mental health is required (11, 59). The European Psychiatric Association (EPA) recommended to implement professional guidelines into practice and harmonize psychiatric clinical practice across Europe to monitor the treatment outcomes of patients with COVID-19 and pre-existing mental disorders; to keep psychiatric services active by using all available options (for example telepsychiatry); to increase communication and cooperation between different health care providers. Anyway, significant differences between countries emerged in service delivery and often traditional face-to-face visits were replaced by online remote consultations (60).

The unpredictability and uncertainty of the COVID-19 pandemic would have required a more sustainable and flexible adaptations of delivery systems for mental health care, even specifically designed to mitigate disparities in health-care provision (61).

Pediatric providers are not always at the forefront of policy negotiations, but they are most certainly on the frontline of the child and family behavioral health crisis. Therefore, pediatric providers can be a powerful influencing policy, with their on the-ground experience caring for families whose well-being has been impacted by the COVID-19 pandemic (62).

## What works according to the evidence

1. The following interventions have been shown to be effective in decreasing mental health problems in children and adolescents (2, 3, 45, 46, 52, 63):
  – community based social support services specifically addressed to mental health problems in children and adolescents with primary care working in partnership with multidisciplinary mental health staff
  – clinician-led mental health and psychosocial services of support in schools and universities
  – positive coping skills, parent-child discussions mediating, art-based programs.

## Recommendations from EAP and ECPCP

The EAP and the ECPCP strongly believe that action is necessary to reduce the negative effects of the pandemic on children's and adolescents' mental health and to advocate for an improved, integrated, and family-focused behavioral health system (11, 42, 58, 62).

### Recommendations to the authorities

– promoting families' supportive interventions and resources in addressing mental health problems in children and young people should be implemented, especially in primary care (11, 15, 53, 59, 62, 63), scaling up the existing mental health support in education and healthcare systems, in close connection with multidisciplinary networks (11, 53, 59, 62)
– adequate funding should be made available for increasing service capacities for the resources we are directing families to contact
– adequate funding should be made available to scientific research on positive and negative long-term effects of COVID-19 pandemic and prolonged social distancing measures on the mental health of children and young people, as well the influence of specific risk factors evolving over time, to guiding future public health policies (59, 62–64)
– information for children and young people and their parents outlining warning signs of mental health problems, how to recognize them and how to find help, should be published (11)

### Recommendations to educational services for children and adolescents

– students support to remain in school and in education should be a priority especially for young people at risk of early school leaving, for comorbidities, low income and/or ethnic minority backgrounds, previous mental health issues or substance use disorders, to avoid disruptions in learning (53, 59)
– mental health support services and information dissemination in schools and universities, with easier access to in-person services, should be urgently resumed (11, 53, 54)
– mental health hotlines by phone or online services providing emergency support to young people should be maintained and adequately funded, whilst further evidence on their effectiveness is being collected (11, 53, 54)
– school administrators should be aware of the resources available for children and young people and their parents outlining warning signs of mental health problems, how to recognize them and how to find help and they should signpost these resources to all of them (11, 52, 53, 59).

### Recommendations to all parents and caregivers

– they should ask for support from primary care professionals to intercept early signals of stress, to improve family well-being and decrease the psychological impact of SARS CoV-2 Pandemic on their children (58, 65, 66), see Table 1

**Table 1:** Recommendations for parents to decrease psychological impact of SARS CoV-2 pandemic.

• Improve family cohesion • Communicate appropriately and talk with your children/adolescents about feelings • Maintain social contacts • Create a secure emotional environment • Keep your children safe from weapons, abuse, and preventable incidents • Improve resilience with adequate support or seek help from professionals

Several open access parenting resources are freely accessible, also on non-smartphones devices, as the Internet of Good Things_IoGT (67).

– they should be aware about the signs or symptoms of anxiety or depression in their children and adolescent (68–70), to look out for and to timely recognize them and seek for professional support. Primary care pediatricians can discuss signs and indicators of mental health concerns with parents and caregivers to increase identification of mental health needs. Guidelines for these discussions are presented in Table 2.

**Table 2:** Suspicious signs or symptoms of anxiety or depression in children and adolescents.

Depression	Anxiety
Sadness or irritability for 2 weeks or more	Problematic fear or worry
Change in functioning	Panic attacks
Change in weight, appetite or sleeping	Avoidance of activities related to fear
Less pleasure in activities with people	Muscle tension symptoms
**Shared symptoms of both depression and anxiety**	
Sleep problems	Changes in thinking
Excessive guilt, preoccupation with errors	Irritability, tantrums, or defiance
Tiredness, low energy	Thoughts of self-harm, or suicide

From: Gleason MM, et al. Modified (70).

### Recommendations to pediatricians

• working together with all the other health care professional with expertise in the field, pediatricians:
  – should actively participate in multidisciplinary networks, including psychologists and psychiatrists, to support parents, children, and adolescents at risk (53, 58, 59)
  – should produce guidelines necessary to policy makers to alleviate the negative effects of Pandemic (11, 53, 59)
  – should continue to work in a holistic and family-oriented manner, exploring both issues related to the physical health and issues related to the psychosocial context (11, 53, 58, 59)
• working together with parents/caregivers and educators, pediatricians:
  – should be aware of the specific resources available for children, adolescents, and their parents, and should also signpost these to them, outlining warning signs (11)
  – should work together with parents and teachers to monitor children's and adolescents' suspect signs of depression and anxiety with specific screening questionnaires, during health care visits in primary care, and treat them with targeted and shared treatment plans, or refer when needed (48, 49, 54, 55, 62)
  – should talk properly with children and adolescents about depression and anxiety issues (11, 70)
  – should monitor over time the duration, evolution, and outcomes of children and adolescents needs and mental health problems (11, 45, 62)
• working together with politicians, pediatricians
  – should campaign to policy makers to create a tailored and integrated net between health and social services, according to resources and prevalence of mental health problems in each European country (11, 42, 45–47, 53, 59)
  – should promote investment policies for enhancing well-being (11, 42, 45–47, 53, 59, 62), improved integration of social and health services in families, in schools or at the community level, especially for high-risk populations (low-income families, comorbidities, previous mental health diseases) (11, 53, 57–59)
  – must inform politicians about the available strategies and treatments needed to help children and adolescents to thwart the pandemic adverse effects (53, 59)
  – must require a specific multiprofessional and integrated medical education on mental health problems management, as well as adequate allocation of funds targeted for this purpose (11, 42, 53, 56, 59).

## Conclusions

While exacerbating negative consequences for mental health, the pandemic also offers us an opportunity to rethink our approach and to build back better by investing in a comprehensive approach to mental health that is fit for the future, as proposed by UNICEF World's Children Report 2021. COVID-19 is not the first microorganism to threaten humanity, and probably will not be the last. We need to utilize effective strategies to strengthen families to respond, care, and protect a future for their children (11) and to promote a comprehensive and strong mental health system policy response (11, 58). This means increasing access to mental health services, expanding, and developing a family-focused mental health workforce, also promoting the integration of mental health in pediatric primary care and improving school's services (62).

All children and adolescents, regardless of their social and health conditions, need and have the rights to safe, secure, inclusive homes, schools, and social environments in which to develop and thrive (11) according to the principles of social justice mentioned in the ISSOP statement (71), following the pediatric integrated care models (72, 73), for mental health also (74, 75), and the Nurturing Care Framework principles (76).

We have a historic chance to commit, communicate and take action to promote, protect and care for the mental health of a generation (11).

## Data Availability

The original contributions presented in the study are included in the article/Supplementary Material, further inquiries can be directed to the corresponding author/s.
